# Diagnostic efficacy of double-balloon enteroscopy in patients with suspected isolated small bowel Crohn’s disease

**DOI:** 10.1186/s12876-020-01188-0

**Published:** 2020-02-26

**Authors:** Zihan Huang, Xiang Liu, Fei Yang, Guoxin Wang, Nan Ge, Sheng Wang, Jintao Guo, Siyu Sun

**Affiliations:** The Department of Gastroenterology, Shengjing Hospital, China Medical University, No.36, Sanhao Street, Shenyang, 110004 Liaoning Province China

**Keywords:** Double-balloon enteroscopy, Inflammatory bowel disease, Crohn’s disease, Small bowel

## Abstract

**Background:**

Owing to the development of double-balloon enteroscopy (DBE) and video capsule endoscopy (VCE) in recent years, direct visualization of the entire small intestinal mucosa has become possible. Because of the nonspecific symptoms and the anatomic location of the small bowel, diagnosis of isolated small bowel Crohn’s disease (CD) remains a challenge. The aim of this research was to explore the value of DBE for isolated small bowel CD in situations where routine tests cannot confirm the diagnosis.

**Methods:**

This study included patients with suspected isolated small bowel CD who were hospitalized in Shengjing Hospital from April 2014 to June 2018. We included patients presenting with chronic diarrhea, abdominal pain, abdominal mass, perianal lesions, and systemic symptoms including weight loss, fever, and anemia after excluding infection factors. Patients with purely colonic CD were excluded from this cohort. Patients with suspected isolated small bowel CD underwent DBE.

**Results:**

In 16/18 patients, pathological findings were detected by DBE. In 12 of the cases, small bowel CD was confirmed. The remaining four patients were diagnosed with small bowel inflammation, duodenal carcinoma, ileum inflammation and small bowel ulcers. However, the diagnosis of CD was confirmed in 14/18 (78%) patients by taking into account the clinical presentation, endoscopic and histological results as well as the experimental treatment. DBE assisted in the diagnosis in 86% (12/14) of the patients.

**Conclusions:**

In the diagnosis of small bowel CD, DBE is a helpful tool. Before assessment with DBE, clinical features, colonoscopy, and CT were used to initially assess the intestine. According to the lesions indicated by CT, we chose the most appropriate endoscope insertion route, and combined the endoscopic characteristics and pathological results of DBE to confirm the diagnosis.

## Background

Crohn’s disease (CD) is an inflammatory disease that can involve the entire gastrointestinal tract. According to previous reports, in 30–70% of patients with CD, the small bowel is affected, and in up to 30% of patients diagnosed with CD, only the small bowel is involved [1, 2]. It is difficult to diagnose isolated small bowel CD because of the nonspecific symptoms and anatomic location of the small bowel. According to the location and pathological behavior (including the development of penetration and strictures), CD has different clinical manifestations. The pathological changes occurring in the distal part of the ileum manifest mainly as intestinal stenosis, which occurs as the disease progresses [3, 4]. Stenosis and penetration (perforation) are major complications and require surgical intervention. Some patients with a long medical history and recurrence of disease complain of obstructive symptoms in the first medical consultation, and many patients receive delayed diagnosis and treatment. Early identification and treatment of CD involving the small bowel may be beneficial to these patients.

As there is no gold standard for the diagnosis of CD, the defined diagnosis usually requires the combination of clinical, endoscopic, radiological, and histological features and the exclusion of an infectious etiology [5]. Traditionally, the tools for diagnosing CD have included esophagogastroduodenoscopy (EGD), colonoscopy, X-ray tests, and high-resolution ultrasound. Computed tomography (CT) and magnetic resonance (MR) are traditionally used standards for examining the small intestine. CT and MR are accurate techniques for detecting extraluminal complications. The European consensus has proposed the use of MR enterography/enteric (MRE) and CT enterography/enteric (CTE) to detect intestinal involvement and penetrating lesions in CD. Both are considered imaging techniques with the highest diagnostic accuracy [6, 7]. Video capsule endoscopy (VCE) and double-balloon enteroscopy (DBE) have become effective tools in diagnosing small bowel CD [8]. The advantage of DBE is not only direct visualization of the small bowel mucosa but it also allows for biopsy of the lesion and therapeutic interventions [9]. DBE can be used to diagnose unclear small bowel disease or suspected cancer and can also be used in rare cases in which tissue pathological examination is required. The aim of this research was to explore the value of DBE for the diagnosis of isolated small bowel CD.

## Methods

This study was conducted in accordance with the Helsinki Declaration and was approved by the ethics committee of Shengjing Hospital of China Medical University. It was conducted at Shengjing Hospital from April 2014 to June 2018. DBE was performed on patients with clinically suspected small bowel CD. The patients’ medical records were retrieved from the prospective hospital database and reviewed for general information, medical history, physical examination, laboratory tests, radiology, endoscopy and histology results. All patients signed a written informed consent to undergo DBE and were informed about the risks of the examination, including the biopsy procedure.

We included patients presenting with chronic diarrhea, abdominal pain, abdominal mass, perianal lesions, and systemic symptoms including weight loss, fever, and anemia after excluding infection factors. Preliminary laboratory tests included routine blood work, C-reactive protein (CRP), erythrocyte sedimentation rate (ESR), serum albumin, and fecal calprotectin. Patients underwent EGD, colonoscopy, CT and additional imaging modalities such as CTE or VCE. For patients with suspected obstruction, before VCE was preformed, it was necessary to confirm the disappearance of the obstruction according to the assessment of results by CT. After analyzing these abnormal findings, we performed DBE in patients with high suspicion of small bowel CD. Patients with colonic lesions were excluded from this study.

All patients were fasted for at least 12 h and polyethylene glycol-based bowel preparation was administered before the procedure. DBE was performed in an endoscopic operating room with fluoroscopic function. Patients were administered conscious sedation with propofol (Lipuro®; Braun, Melsungen, Germany) by an anesthesiologist and then subjected to electrocardiographic monitoring. DBE was performed using Fujinon enteroscopes (Fujinon EN 450P 5/20, EN-450 T5; Fujinon Corp, Saitama, Japan). Carbon dioxide (CO_2_) was pumped using a CO_2_ regulator (Olympus UCR; Olympus) connected to a CO_2_ gas cylinder during the procedure. The flow rate for CO_2_ insufflation was set at 1.0 L/minute in all patients. The TCM4 detector (Linde Medical Sensors, Basel, Switzerland) used a low-pressure clip connected sensor to measure PtcCO_2_ non-invasively and continuously. PtCO_2_ was measured just before the examination and after finishing the procedure. We used biopsy forceps to perform tissue acquisition of suspected lesions in different sites. At least two samples were taken from each site and five samples were taken at the site of obvious lesions.

## Results

A total of 49 patients were diagnosed with small bowel CD, of which 18 patients were difficult to confirm by conventional tests. 13 males and 5 females were included in this study and 21 DBEs were performed. The mean age of patients diagnosed with small bowel CD was 41.5 years. In seven patients, the insertion route was oral, in eight patients the insertion was through the anal route, and in three patients both oral and anal approaches were used. We estimated insertion depth on the basis of the number of strokes (insertion and withdrawal cycles). The average depth of insertion was 235 ± 95 cm (oral route) and 77 ± 40 cm (anal route). Clinical characteristics of the patients, endoscopic findings and CT results are summarized in Table 1.

**Table 1:** Results of EGD, colonoscopy, CT in patients with suspected isolated CD of the small bowel

No.	Age ranges	Indication	EGD	Colonoscopy	CT	Additional imaging
1	50–60	Abdominal pain, diarrhea	Without pathological findings	Ileum terminal congestion and erosion	Thickening of the ileum wall	
2	< 16	Abdominal pain	Esophagitis	Without pathological findings	Thickening of jejunum wall, low intestinal obstruction	VCD: Without pathological findings CTE: Thickening of jejunum wall
3	50–60	Abdominal pain	Without pathological findings	Colonic mucosal congestion and edema	Thickening of jejunum wall, mild luminal narrowing	
4	16–20	Abdominal pain, hypodynamia	Without pathological findings	Ileum terminal congestion	Thickening of the ileum wall	VCE: Ulcerations in the ileum (H1-S2) CTE: Ileum mural hyper-enhancement and bowel wall thickening
5	60–70	Abdominal pain	Without pathological findings	Polyp	Thickening of the ileum wall	
6	50–60	Abdominal pain, melena	Gastritis	Two polyps	Thickening of jejunum wall	VCE: Ulcerations in the ileum
7	50–60	Vomiting	Esophagitis, gastritis	Without pathological findings	Thickening of duodenum wall	
8	20–30	Abdominal pain, perianal pain	Esophagitis	Ileum terminal congestion and lymphoid follicular hyperplasia	Thickening of the ileum wall	CTE: Perianal fistula, thickening of the ileum wall, mural stratification and hyper-enhancement
9	20–30	Abdominal pain	Without pathological findings	Colonic congestion and erosion	Thickening of the ileum wall	CTE: Thickening of the ileum wall, mural stratification and hyper-enhancement, increased attenuation of the mesenteric fat
10	40–50	Melena	Without pathological findings	Ileum terminal congestion	Thickening of jejunum wall	VCE: Ulcerations in the junction of jejunum and ileum CTE: Jejunum mural hyper-enhancement and bowel wall thickening
11	50–60	Abdominal pain	Gastritis	Without pathological findings	Thickening of jejunum wall	CTE: Thickening of jejunum wall
12	60–70	Melena, hypodynamia	Without pathological findings	Without pathological findings	Thickening of ileum wall	VCE: Ulcerations in the ileum
13	20–30	Abdominal pain, perianal pain, diarrhea	Without pathological findings	Colonic congestion and erosion	Perianal fistula, abscesses, thickening of the ileum wall	CTE: Perianal fistula and thickening of the ileum wall
14	60–70	Abdominal pain	Without pathological findings	Rectal congestion and edema	Thickening of the ileum wall	
15	20–30	Abdominal pain, diarrhea	Gastritis, duodenitis	Without pathological findings	Suspected intestinal obstruction	VCE: Without pathological findings
16	20–30	Abdominal pain, diarrhea	Duodenitis	Without pathological findings	Perianal fistula, thickening of the intestinal wall	CTE: Perianal fistula, multi-segment mural hyper-enhancement
17	60–70	Nausea, vomiting, diarrhea	Gastric ulcer	Without pathological findings	Occupying lesion of jejunum	
18	20–30	Abdominal pain	Without pathological findings	Without pathological findings	Thickening of the ileum wall	VCE: Ulcerations in the jejunum, luminal narrowing CTE: Ileum mural hyper-enhancement and bowel wall thickening

*CD* Crohn’s disease, *CT* Computed tomography, *EGD* Esophagogastroduodenoscopy, *VCE* Video-capsule endoscopy, *CTE* Computed tomography enterography/enteroclysis

**Fig. 1:**
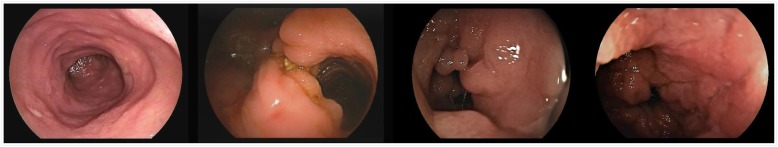
Endoscopic performance of small bowel Crohn’ s disease under double balloon enteroscopy

The endoscopic features suggesting a diagnosis of CD include aphthous ulcers, longitudinal ulcers, a cobblestone-like appearance, intestinal stenosis and segmental lesions, as shown in Fig. 1. Microscopic characteristics of CD in biopsy specimens are focal (discontinuous) chronic inflammation, focal crypt irregularity (discontinuous crypt distortion), and granulomas (not related to crypt injury). Aphthous ulcers, linear ulcers, deep fissures with knife-like clefts, and transmural lymphoid hyperplasia also suggest a CD diagnosis. The results of DBE, histological findings and clinical diagnoses are summarized in Table 2.

**Table 2:** Results of DBE and histological examination

No.	Insertion route	Insertion depth (cm)	DBE	Biopsy	Clinical diagnosis
1	Anal	70	Mucosal edema, congestion and erosion, multiple polypoid hyperplasia	Focal chronic inflammation, crypt regularity	Ileum inflammation
2	Oral	330	Multiple longitudinal ulcers in jejunum and ileum, cobblestone-like appearance	Focal chronic inflammation, lymphoid hyperplasia	CD (A1, L4, B2)
2^	Anal	40	Without pathological findings	No data	CD (A1, L4, B2)
3	Oral	300	Intestinal stenosis, segmental ulcers in the junction of jejunum and ileum	Focal inflammation, ulcerative lesions	CD (A3, L4, B2)
4	Anal	40	Multiple aphthous ulcers and longitudinal ulcers	Focal chronic inflammation, crypt irregularity and lymphoid hyperplasia	CD (A2, L1, B1)
5	Oral	160	Without pathological findings	No data	CD (A3, L4, B1)
6	Oral	350	Multiple aphthous ulcers in jejunum	Ulcerative lesions, mild dysplasia	Small bowel ulcers
7	Oral	20	Intestinal stenosis, longitudinal ulcers	Focal high-grade intraepithelial neoplasia	Duodenal carcinoma
8	Anal	30	Multiple longitudinal ulcers in ileum	Granulomas, acute inflammation and focal crypt irregularity	CD (A2, L1 + L4, B1p)
9	Anal	75	Intestinal stenosis, longitudinal ulcers in ileum	Focal chronic inflammation, lymphoid hyperplasia	CD (A2, L4, B2)
10	Oral	240	Without pathological findings	No data	CD (A3, L1 + L4, B1)
11	Oral	240	Mucosal congestion and erosion	No data	Small bowel inflammation
12	Anal	30	Aphthous ulcers, mucosal congestion	Focal acute and chronic inflammation, lymphoid hyperplasia	CD (A3, L4, B1)
13	Anal	85	Segmental lesions of longitudinal ulcers in ileum, cobblestone-like appearance	Focal chronic inflammation, focal crypt irregularity	CD (A2, L1 + L4, B1p)
14	Anal	90	Multiple ulcers and ulcer scars in ileum, polypoid hyperplasia	Focal acute and chronic inflammation	CD (A3, L4, B1)
15	Anal	140	Segmental lesions of longitudinal ulcers in ileum	Adenomatous hyperplasia with lymphoid hyperplasia	CD (A2, L4, B2)
16	Anal	140	Aphthous ulcers	No data	CD (A2, L4, B2p)
16^	Oral	270	Intestinal stenosis, longitudinal ulcers in jejunum	Ulcer with mild atypical hyperplasia, focal acute and chronic inflammation	CD (A2, L4, B2p)
17	Oral	190	Intestinal stenosis, multiple longitudinal ulcers in jejunum	Focal acute and chronic inflammation, focal crypt irregularity	CD (A3, L4, B2)
18	Anal	110	Without pathological findings	No data	CD (A2, L4, B2)
18^	Oral	250	Intestinal stenosis, segmental ulcers	Focal inflammation, crypt irregularity and transmural lymphoid hyperplasia,	CD (A2, L4, B2)

*CD* Crohn’s disease, *DBE* Double balloon enteroscopy, 2^, 16^, 18^: Contrary to the first direction

**Fig. 2:**
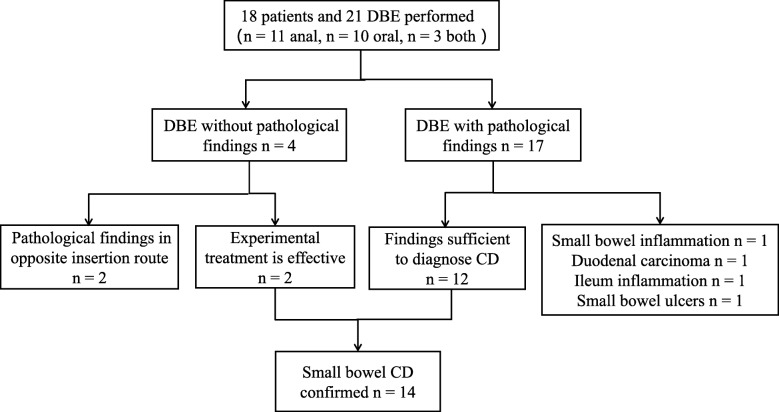
Diagnostic work flow of 18 patients with suspected isolated small bowel Crohn’ s disease (CD). DBE: double-balloon enteroscopy; VCD: video capsule endoscopy; CT: computed tomography

As shown in Fig. 2, in 18 patients, 16 had abnormal lesions observed via DBE, and 12 were diagnosed with small bowel CD after comprehensive analysis. Among the remaining four patients, one was diagnosed with small bowel inflammation, one was diagnosed with duodenal carcinoma, one was diagnosed with ileum inflammation, and one was diagnosed with small bowel ulcers. Of all patients enrolled, only two patients were observed to have no abnormal lesions via DBE, but were later clinically confirmed to have small bowel CD after experimental treatment. The principle of DBE operation is to insert the scope into the deep small bowel as much as possible. In one patient, DBE terminated in the middle of the jejunum, with an insertion depth of 240 cm, and the patient’s VCE showed multiple ulcerative lesions in the lower part of the jejunum and the upper part of the ileum. In another patient, DBE reached the upper part of the jejunum, with a depth of 160 cm through the oral side, until further insertion was not possible.

After the DBE results were combined with the clinical presentation of patients and imaging findings and experimental treatment, a diagnosis of CD was confirmed in 14 patients (78%). DBE assisted in diagnosis in 86% (12/14) of the patients.

## Discussion

Owing to the characteristics of the small bowel anatomy, a sensitive and specific tool for the diagnosis of small bowel disease has been lacking. In our study, we found that DBE was useful to diagnose or confirm small bowel CD in patients after exclusion of abnormal changes in the upper gastrointestinal tract and colon. The absence of a gold standard for diagnosis also makes confirming small bowel CD difficult. For diagnosis of small bowel CD, many new techniques can be used to identify small bowel lesions [6, 10]. Recent studies have shown that the imaging techniques MRE and CTE have high sensitivity and specificity for recognizing active inflammation in the small bowel, especially for identifying stenosis, penetration, and extra-intestinal manifestations. On CTE, enteric findings such as mural hyper-enhancement, bowel wall thickening, mural stratification, engorged vasa recta (“comb sign”), and increased attenuation of the mesenteric fat are features of active inflammatory small bowel CD. According to the literature, the most common manifestation of CTE in established CD patients is thickening of the intestinal wall, up to at least 80% [11], in agreement with our results. Although this feature is not characteristic, the abnormalities in CTE should be different from those in cryptogenic multifocal ulcerous stenosing enteritis, intestinal involvement of diffuse connective tissue disease, and chronic ischemic bowel disease. CTE can indicate the location and extent of the lesion, providing clues to direct the insertion direction of DBE. Their noninvasiveness makes CTE and MRE acceptable and feasible in clinical practice [7, 12].

VCE is a noninvasive method with high sensitivity for detecting small bowel lesions. It can enable visualization of mucosal lesions, particularly superficial lesions [13, 14]. Lesions detected by VCE are nonspecific, and CD cannot be distinguished from lesions caused by nonsteroidal anti-inflammatory drugs (NSAIDs) or other etiologies, on the basis of endoscopic images [15]. The randomness of the image capture in VCE results in high negative predictive value and lower specificity [16]. Positive consequences must be further identified and confirmed. Failure to obtain tissue for pathological results and the potential risk of capsule retention are major disadvantages of VCE. DBE is superior to radiology for detecting superficial lesions such as aphthous ulcers and erosions. Compared with VCE, it can accurately describe the location and morphology of lesions and surrounding mucosa (such as the appearance of pebbles and edema), thus helping to distinguish CD from other diseases [17, 18].

DBE is a helpful method when no abnormalities are observed using radiology and VCE but small bowel CD is suspected. Since the development of DBE in 2001 by Yamamoto et al. [9], its diagnostic ability in small bowel diseases has been extensively studied. It allows for direct visualization of the small bowel mucosa and has a broader and clearer field of vision than VCE [19]. CD is characterized by chronic granulomatous intestinal inflammation, and the lesions tend to be segmental and discontinuous. Multifocal lesions may have areas of different activity, and acute inflammatory and fibrotic strictures may be present at the same time. DBE can be used to visualize large portions of the small bowel, alone or in combination, and to examine small bowel inflammatory lesions [20]. The earliest and most characteristic endoscopic finding in small bowel CD is aphthous ulcers, which enlarge and deepen as the disease progresses, thus forming longitudinal ulcers. CD inflammation is often discontinuous, bordering on normal tissue, thereby resulting in segmental lesions. A cobblestone-like appearance occurs when longitudinal ulcers pass through areas of normal or inflamed tissue [5]. Other common endoscopic findings are thickening of the intestinal wall, varying degrees of stenosis, and cluster-like polyposis. The morphological features detected by DBE play a crucial role in the diagnosis of CD. A longitudinal ulcer along the mesenteric side is a typical morphological feature that can help to distinguish CD from other diseases that cause intestinal inflammation [21]. Ulcers associated with ischemia, intestinal tuberculosis, and Behcet’s disease are common on the anti-mesenteric side of the bowel, whereas NSAID-associated ulcers have no such tendency in the intestinal lumen. The combination of typical macroscopic features with clinical manifestation, laboratory tests, smoking status, and family history may be sufficient to diagnose CD.

Ideally, DBE can provide pathological support for the diagnosis of small bowel CD [22, 23]. The histology usually detected in patients with established CD includes focal (discontinuous) chronic inflammation, focal crypt irregularities, and granulomas [6]. Non-caseous granulomas are generally recognized as the most important microscopic features detectable for the diagnosis of CD. Non-caseous granuloma and at least one other microscopic feature (focal chronic inflammation or focal crypt irregularity) can be considered for CD [24]. In a retrospective study [22], despite positive macroscopic findings from DBE, 58% of the patients had normal or nonspecific histology, and 45% of patients were treated as having CD on the basis of a combination of histology, endoscopic appearance, clinical symptoms and laboratory tests. Although the discontinuity of inflammation and the superficiality of the biopsy tissue results in a lower chance of obtaining a granuloma under endoscopy, 10–30% of histological findings can still provide evidence to establish a diagnosis [25]. Clinical diagnosis requires pathological support to confirm the diagnosis of small bowel CD and to help differentiate it from intestinal tuberculosis, Behcet’s disease, lymphoma, and other diseases. Small bowel CD can be diagnosed using the six diagnostic points proposed by the WHO [24]. The diagnosis of small bowel CD involves clinical, endoscopic, radiological, and histological features; however, satisfying all the criteria may be impossible or unnecessary in practice. DBE should be considered complementary to other diagnosis methods. In ambiguous cases, the “test of time” is useful for CD diagnosis. Recurrent complaints, responses to therapy, and recurrent symptoms after stopping therapy may eventually verify the diagnosis.

When DBE presents as only superficial ulcers and mucosal inflammation in the small bowel, patients cannot be sufficiently diagnosed with CD. Studies have reported that using endoscopic ultrasound (EUS) with DBE is more helpful in diagnosing inflammatory bowel disease because it shows the hierarchical structure of the intestinal wall of the small bowel [26, 27]. Very few studies have reported the application of EUS with DBE in the small bowel, but EUS has been widely used in the diagnosis of digestive tract disease [28–31]. In addition to aiding in the diagnosis of small bowel CD, DBE can be useful in the provision of therapeutic interventions. Retrieval of retained VCE devices has been reported in several studies [8, 32]. Owing to the recurrent nature of the disease, patients are prone to intestinal stenosis, and many patients will require multiple surgeries during the disease course. Surgical removal of the stenosis is a recognized solution, and half of patients require surgery within the first decade of diagnosis. A meta-analysis [33] has reported that small bowel clinical recurrence occurs in approximately 28% of patients after total colectomy with permanent ileostomy for colonic CD. Endoscopic balloon dilation (EBD) is a safe and effective method that can replace small bowel resection in some cases [34].

In our study, no adverse events occurred (e.g., bleeding, perforation, pancreatitis and sedative-related adverse events) after DBE. According to different reports, the incidence of DBE complications is between 1.2% and 1.6% [35, 36]. When the small bowel is contorted, or stenosis is present, any further insertion of the endoscope would increase the risk of perforation. For this reason, we stopped insertion in such situations. Determination of the most appropriate DBE insertion route is based on lesion location information provided by CT or other additional examinations. For the seven patients who underwent VCE, their insertion route was based mainly on the results of VCE. When the VCE and CT results were very different or without clear evidence of small bowel involvement, we chose the oral route for insertion because deeper insertion can be achieved via the oral route, increasing the potential of finding the lesions [37]. According to Mays’ method [38], which is widely used in clinical practice and has been found to be effective in estimating the depth of endoscopic insertion, we estimated insertion depth by calculating the number of strokes (insertion and withdrawal cycles).

There are several limitations in this study that must be discussed. First, the study was a retrospective study with a small sample of patients. Because of the low incidence and low prevalence of small bowel CD in Asian regions, in this single center study, we were unable to obtain a larger sample, which may have affected diagnostic accuracy. Second, because skilled endoscopists and anesthesiologists are required, the application of DBE in the diagnosis of small bowel CD is currently not widely used. Because our hospital is a tertiary referral center, our patients may not be representative of patients with small bowel CD. Many of the patients in this study had a relatively long medical history, and their condition was quite serious; thus, these patients may have had typical and obvious macroscopic features, enabling easier detection of lesions via DBE. On the one hand, this may have increased the diagnostic accuracy of DBE; on the other hand, opportunities to identify lesions may have been missed because of complicated abdominal conditions that limited the use of DBE, such as surgical adhesion and deep ulcers or stenosis of the bowel.

## Conclusions

In patients with suspected small bowel CD, we recommend the use of DBE for the comprehensive evaluation of the gastrointestinal tract, thus contributing to clinical diagnosis in cases of negative EGD and colonoscopy. DBE is suitable when VCE or radiological examination reveals abnormal lesions, or when the results of these two methods are negative but small bowel CD is highly suspected. In the future, more indications using DBE will be developed to diagnose, monitor and treat small bowel CD.

## Data Availability

The datasets used and/or analysed during the current study available from the corresponding author on reasonable request.

## Abbreviations

CD: Crohn’s disease
CO2: Carbon dioxide
CRP: C-reactive protein
CT: Computed tomography
CTE: Computed tomography enterography/enteroclysis
DBE: Double-balloon enteroscopy
EBD: Endoscopic balloon dilation
EGD: Esophagogastroduodenoscopy
ESR: Erythrocyte sedimentation rate
EUS: Endoscopic ultrasound
MRE: Magnetic resonance enterography/enteroclysis
NSAIDs: Nonsteroidal anti-inflammatory drugs
VCE: Video capsule endoscopy
